# Microbiological Quality Assessment of Chicken Thigh Fillets Using Spectroscopic Sensors and Multivariate Data Analysis

**DOI:** 10.3390/foods10112723

**Published:** 2021-11-07

**Authors:** Evgenia D. Spyrelli, Christina K. Papachristou, George-John E. Nychas, Efstathios Z. Panagou

**Affiliations:** Laboratory of Microbiology and Biotechnology of Foods, Department of Food Science and Human Nutrition, School of Food and Nutritional Sciences, Agricultural University of Athens, Iera Odos 75, 11855 Athens, Greece; eugeniespcheng@gmail.com (E.D.S.); kristpap@hotmail.com (C.P.); gjn@aua.gr (G.-J.E.N.)

**Keywords:** poultry meat, spoilage, multispectral imaging, Fourier-Transform Infrared spectroscopy (FT-IR), regression models, classification models, multivariate data analysis

## Abstract

Fourier transform infrared spectroscopy (FT-IR) and multispectral imaging (MSI) were evaluated for the prediction of the microbiological quality of poultry meat via regression and classification models. Chicken thigh fillets (*n* = 402) were subjected to spoilage experiments at eight isothermal and two dynamic temperature profiles. Samples were analyzed microbiologically (total viable counts (TVCs) and *Pseudomonas* spp.), while simultaneously MSI and FT-IR spectra were acquired. The organoleptic quality of the samples was also evaluated by a sensory panel, establishing a TVC spoilage threshold at 6.99 log CFU/cm^2^. Partial least squares regression (PLS-R) models were employed in the assessment of TVCs and *Pseudomonas* spp. counts on chicken’s surface. Furthermore, classification models (linear discriminant analysis (LDA), quadratic discriminant analysis (QDA), support vector machines (SVMs), and quadratic support vector machines (QSVMs)) were developed to discriminate the samples in two quality classes (fresh vs. spoiled). PLS-R models developed on MSI data predicted TVCs and *Pseudomonas* spp. counts satisfactorily, with root mean squared error (RMSE) values of 0.987 and 1.215 log CFU/cm^2^, respectively. SVM model coupled to MSI data exhibited the highest performance with an overall accuracy of 94.4%, while in the case of FT-IR, improved classification was obtained with the QDA model (overall accuracy 71.4%). These results confirm the efficacy of MSI and FT-IR as rapid methods to assess the quality in poultry products.

## 1. Introduction

Food waste amounts to 14% of the worlds’ food consumption [1], while meat and specifically poultry production is forecasted to rise at 137 million tones [2]. In addition, consumer’s awareness and demand for high quality and safety meat and poultry has been continuously increased. For this purpose, non-invasive spectroscopic sensors have been used in the evaluation of the quality and freshness of meat products [3] through the implementation of process analytical technology (PAT) [4,5]. The underlying principle of PAT is to combine spectral data acquired through real-time (in-, on-, at-line) non-destructive analytical techniques with multivariate data analysis for the development of models assessing food quality. These models, along with their datasets, could be uploaded in the cloud, updated regularly with new data in order to be consultative to the food industry [6].

In recent years, multispectral imaging (MSI) and Fourier transform infrared (FT-IR) spectroscopy have been investigated as alternative methods for the evaluation of a variety of meat products [7,8,9]. The former method is a merge of UV and NIR with computer vision, and it has been proposed as an ecological approach for rapid quality evaluation of meat and poultry [10,11,12]. Until now, spectral data in the visible and near-infrared region (400–1700 nm) have been employed in the development of quantitative or qualitative models for the determination of the bacterial population (TVCs and *Pseudomonas* spp.) on chicken meat during spoilage [13,14,15]. In the same context, MSI analysis has been proved a solution to the identification of adulteration/food fraud of minced beef with chicken meat [16], as well as for the detection of food fraud in minced pork adulterated with chicken [17]. Moreover, fecal contaminants in poultry line [18] and the presence of tumors on the surface of chicken breasts [19] have been accurately detected via MSI analysis. This innovative method was successfully employed in the at-line estimation of the time from slaughter in four different poultry products [20].

Likewise, the potential of FT-IR for the qualitative and quantitative assessment of the microbiological quality of meat products has been explored by other researchers [21,22,23,24,25]. Especially for poultry, FT-IR was recommended as an effective approach for the differentiation of intact chicken breast muscle during spoilage [22]. Additionally, the level of spoilage bacteria on the surface of chicken meat was successfully estimated via FT-IR spectroscopy [21]. Further investigation of this promising method for real-time evaluation of the freshness of stored chicken breast fillets was undertaken by Vansconcelos et al. [26]. FT-IR analysis was also proposed as an efficient approach for the categorization of chicken meat among seven raw types of food, irrespective of variations among batches and storage conditions (temperature, storage duration, packaging, spoilage levels) [3].

Spectral data acquired by nondestructive methods such as MSI and FT-IR have been analyzed by a variety of unsupervised and supervised machine learning algorithms for the rapid quality assessment in food matrices including meat [25,27,28]. Partial least squares regression (PLS-R), linear discriminant analysis (LDA), and quadratic discriminant analysis (QDA) have been reported as reliable tools for the development of predictive models for spoilage or adulteration assessment in meat [9,29,30,31]. Moreover, deep learning methodologies such as artificial neural networks (ANNs) and support vector machines (SVMs) [32] have been employed, validated, and compared through available websites (e.g., sorfML, Metaboanalyst) or softwares (R, MatLab, Python), in an attempt to provide accurate quantitative and qualitative models for food spoilage assessment [28,33,34,35,36,37].

The aim of the present work was to develop and evaluate machine learning regression (PLS-R) and classification models (LDA, QDA, SVMs, QSVMs) based on MSI and FT-IR spectral data for the evaluation of the microbiological quality of chicken thigh fillets. More specifically, PLS-R models were developed for the prediction of the microbiota of TVCs and *Pseudomonas* spp. on the surface of chicken thigh, whereas LDA, QDA, SVMs, and QSVMs models were employed for the classification of samples in two quality classes (fresh or spoiled) based on the outcome of sensory analysis. The challenging task in this study was not confined in model development, batch variation and different storage temperatures, but it also considered external validation using two different dynamic temperature profiles simulating temperature scenarios during transportation and storage in retail outlets.

## 2. Materials and Methods

### 2.1. Experimental Design

Three hundred and thirty (330) chicken thigh fillets (ca. 90–110 g/fillet) enclosed in plastic packages (dimensions = 25 cm (width), 90 μm (thickness), permeability ca. 25, 90, and 6 cm^3^ m^−2^day^−1^bar^−1^ at 20 °C and 50% RH for CO_2_, O_2_, and N_2_, respectively) were obtained from a poultry industry in Greece and stored aerobically at eight isothermal conditions (0, 5, 10, 15, 20, 25, 30, and 35 °C). Two independent experiments were undertaken at all isothermal conditions using 4 different batches of chicken meat. Moreover, 72 samples were stored at two dynamic temperature profiles (profile 1 = 12 h at 5 °C, 8 h at 10 °C, and 4 h at 15 °C; profile 2 = 12 h at 0 °C, 8 h at 5 °C, and 4 h at 10 °C), simulating temperature scenarios that can be observed during transportation and storage in retail outlets [38]. At pre-determined time intervals, packages were subjected to microbiological analyses for the enumeration of total viable counts (TVCs) and *Pseudomonas* spp., in parallel with MSI and FT-IR spectral data acquisition. At each sampling point, duplicate packages per isothermal storage condition and triplicate packages from each dynamic temperature profile were subjected to the abovementioned analyses. In addition, chicken samples were subjected to sensory evaluation by a 14-member sensory panel to categorize the samples in two quality classes, namely fresh and spoiled as detailed below. Microbiological counts and sensory scores were correlated with spectral data in order to develop quantitative and qualitative models assessing chicken thigh’s microbial loads (TVCs, *Pseudomonas* spp.) as well as their quality class (fresh-spoiled).

### 2.2. Microbiological Analysis and Sensory Evaluation

A total surface of ca. 20 cm^2^ (four slices of ca. 5 cm^2^ each with a maximum thickness of 2 mm) from chicken thigh fillet was removed aseptically, by means of a sterile stainless steel cork borer (2.5 cm in diameter), scalpel and forceps, added in 100 mL of sterile quarter strength Ringer’s solution (Lab M Limited, Lancashire, UK) and homogenized in a Stomacher device (Lab Blender 400, Seward Medical, UK) for 120 s at room temperature [39]. The microbial load on the surface of chicken was enumerated using serial decimal dilutions in the same Ringer’s solution and 0.1 mL of the appropriate dilution was spread on the following growth media: (a) Tryptic glucose yeast agar (Plate Count Agar, Biolife, Milan, Italy) for the determination of total viable counts (TVCs) incubated at 25 °C for 72 h; (b) *Pseudomonas* agar base (LAB108 supplemented with selective supplement Cetrimide Fucidin Cephaloridine, Modified C.F.C. X108, LABM) for the determination of presumptive *Pseudomonas* spp. incubated at 25 °C for 48 h. The results were logarithmically transformed and expressed as log CFU/cm^2^.

In parallel, sensory evaluation was performed by a 14 member in-house trained sensory panel. For this purpose, samples (*n* = 103) were placed in sterile petri dishes and scored according to their odor using a 3-point hedonic scale as follows—1 = fresh, 2 = acceptable, 3 = spoiled [40]. Samples with scores < 2 were characterized as fresh (Class 1) whereas samples with scores ≥ 2 as spoiled (Class 2). Finally, the sensory outcome was correlated with spectral data in order to assess the quality class of the samples directly from the acquired MSI and FT-IR spectra.

### 2.3. Spectra Acquisition

Multi-spectral images (MSI) were captured using a Videometer-Lab instrument (Videometer A/S, Herlev, Denmark) that acquires images in 18 different non-uniformly distributed wavelengths from UV (405 nm) to short wave NIR (970 nm), namely, 405, 435, 450, 470, 505, 525, 570, 590, 630, 645, 660, 700, 850, 870, 890, 910, 940, and 970 nm. Detailed information about this spectroscopic sensor is provided elsewhere [41]. Each sample corresponded to spatial and spectral data of size m × n × 18 (where m×n is the image size in pixels) [42]. Furthermore, canonical discriminant analysis (CDA) was employed as a supervised transformation building method to divide the images into regions of interest (ROI) using the Videometer-Lab version 2.12.39 software (Videometer A/S, Herlev, Denmark). The final outcome of this segmentation process for each image was a dataset of spectral data including the average value and the standard deviation of the intensity of the pixels within the ROI at each wavelength.

FT-IR data were obtained using an FT-IR-6200 JASCO spectrometer (Jasco Corp., Tokyo, Japan) and a ZnSe 45 HATR (horizontal attenuated total reflectance) crystal (PIKE Technologies, Madison, WI, USA) with a refractive index of 2.4 and a depth of penetration of 2.0 μm at 1000 cm^−1^. Spectra measurements were performed using Spectra Manager Code of Federal Regulations (CFR) software version 2 (Jasco Corp., Tokyo, Japan) in the wavenumber range of 4000–400 cm^−1^, by accumulating 100 scans with a resolution of 4 cm^−1^ and a total integration time of 2 min.

### 2.4. Data Pre-Processing and Analysis

MSI spectral data were pre-processed by baseline offset treatment [43,44] for the development of PLS-R models in order to reduce random or systematic variations and simultaneously improve image resolution [45]. Likewise, for the development of the classification models, MSI data were subjected to standard normal variate (SNV) transformation prior to analysis [46]. Model training was undertaken with the dataset obtained from the storage experiments at isothermal conditions (*n* = 330), where 142 (43.1%) and 188 (56.9%) of the samples were defined as fresh (Class 1) and spoiled (Class 2), respectively. Model optimization was based on leave-one-out full-cross validation (LOOCV) process for PLS-R models and k-fold validation (k = 5) for the classification models. Moreover, the efficacy of the developed models to assess the quality of chicken samples was evaluated by external validation using independent datasets from the two dynamic temperature scenarios (*n* = 72; Class 1 = 36 samples, 50%; Class 2 = 36 samples, 50%).

**Figure 1:**
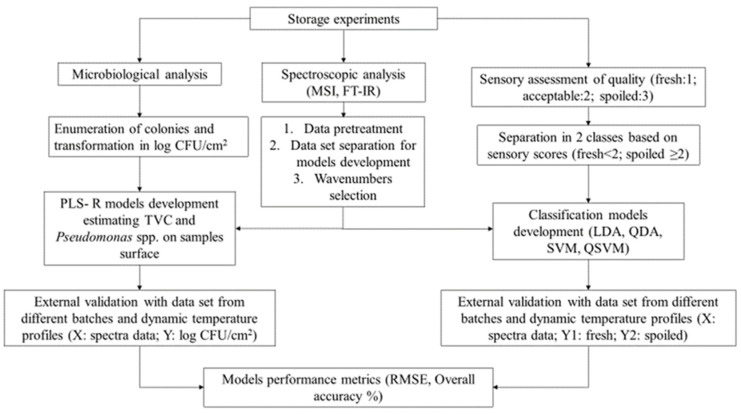
Flowchart describing quantitative and qualitative model development and validation.

FT-IR spectral data were modified by Savitzky-Golay first derivative (second polynomial order, 11-point window) for the development of PLS-R models, while for classification models’ spectral data pre-treatment was based on the same model with a 9-point window in order to reduce baseline shift and noise [9]. Spectral data in the range of 1000 to 2000 cm^−1^ were included in the analysis, since these regions are documented as relevant to meat spoilage [37]. FT-IR models were also validated with data sets from dynamic temperature profiles (*n* = 63), including 30 (47.6%) fresh and 33 (52.4%) spoiled samples. The procedure of model training and validation is graphically presented in Figure 1.

PLS-R models for the estimation of TVCs and *Pseudomonas* spp. counts on chicken thighs surface were developed and validated by the software Unscrambler © ver. 9.7 (CAMO Software AS, Oslo, Norway). Moreover, linear discriminant analysis (LDA) [47], quadratic discriminant analysis (QDA) [31], support vector machines (SVMs), and quadratic support vector machines (QSVMs) [48] models were employed for the classification of samples according to their spoilage level using MATLAB 2012a software (The MathWorks, Inc., Natick, MA, USA). The performance of the developed models was evaluated via the following metrics and indexes: root mean squared error (RMSE), correlation coefficient (r), overall accuracy, sensitivity, and specificity [49,50].

## 3. Results and Discussion

### 3.1. Microbiological Analysis and Sensory Evaluation

**Figure 2:**
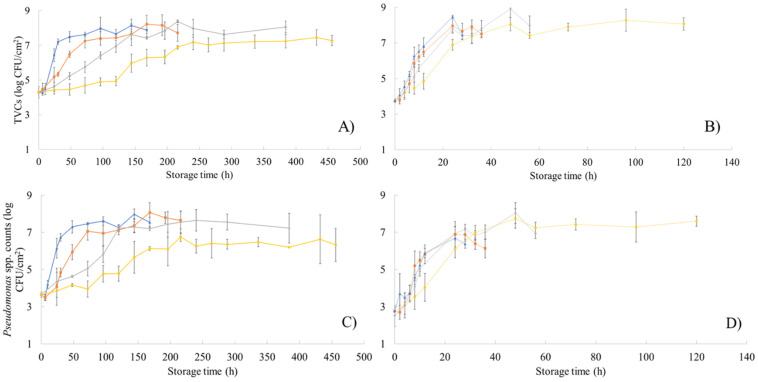
Changes in the population (log CFU/cm^2^) of total viable counts (TVCs) (**A**,**B**) and *Pseudomonas* spp. (**C**,**D**) in chicken thigh samples during storage at different isothermal conditions (**A**,**C**: 0, 5, 10 and 15 °C; **B**,**D**: 20, 25, 30, and 35 °C). Data points are average values of four replicates of samples ± standard deviation.

The population dynamics of TVCs and *Pseudomonas* spp. on the surface of chicken thigh fillets stored at isothermal conditions (0, 5, 10, 15, 20, 25, 30, and 35 °C) are presented in Figure 2. The initial population of TVC (Figure 2A,B) and *Pseudomonas* spp. (Figure 2C,D) was 4.02 (±0.38) and 3.75 (±0.11) log CFU/cm^2^, respectively, confirming previous literature findings [22,51,52]. As expected, storage temperature significantly influenced microbial growth resulting in sample deterioration and spoilage. For poultry, TVCs values exceeding 7.0 log CFU/cm^2^ have been reported by other researchers to signify the end of shelf-life due to spoilage [52,53,54]. More specifically, in this study TVCs reached values above 7.0 log CFU/cm^2^ at 15 °C in 30 h (7.2 ± 0.15 log CFU/cm^2^), at 10 °C in 72 h (7.24 ± 0.39 log CFU/cm^2^), at 5 °C in 144 h (7.62 ± 0.63 log CFU/cm^2^) and at 0 °C in 240 h (7.17 ± 0.42 log CFU/cm^2^). *Pseudomonas* spp. counts were similar to TVCs population and spoilage was evident at 15 °C in 48 h (7.3 ± 0.33 log CFU/cm^2^), at 10 °C in 72 h (7.06 ± 0.48 log CFU/cm^2^), at 5 °C in 120 h (7.22 ± 0.18 log CFU/cm^2^), and at 0 °C in 216 h (6.75 ± 0.23 log CFU/cm^2^). Furthermore, samples appearance and odor rapidly deteriorated at high storage temperatures and TVCs reached 7.0 log CFU/cm^2^ at 20 °C in 32 h (7.36 ± 0.39 log CFU/cm^2^), at 25 °C in 24 h (7.78 ± 0.18 log CFU/cm^2^), at 30 °C in 24 h (7.95 ± 0.40 log CFU/cm^2^), and at 35 °C in 12 h (6.8 ± 0.46 log CFU/cm^2^). Similarly, *Pseudomonas* spp. approached 7.0 log CFU/cm^2^ at 20 °C in 32 h (6.97 ± 0.39 log CFU/cm^2^), at 25 °C in 24 h (6.95 ± 0.36 log CFU/cm^2^), at 30 °C in 24 h (6.89 ± 0.68 log CFU/cm^2^), and at 35 °C in 24 h (6.69 ± 0.60 log CFU/cm^2^).

**Figure 3:**
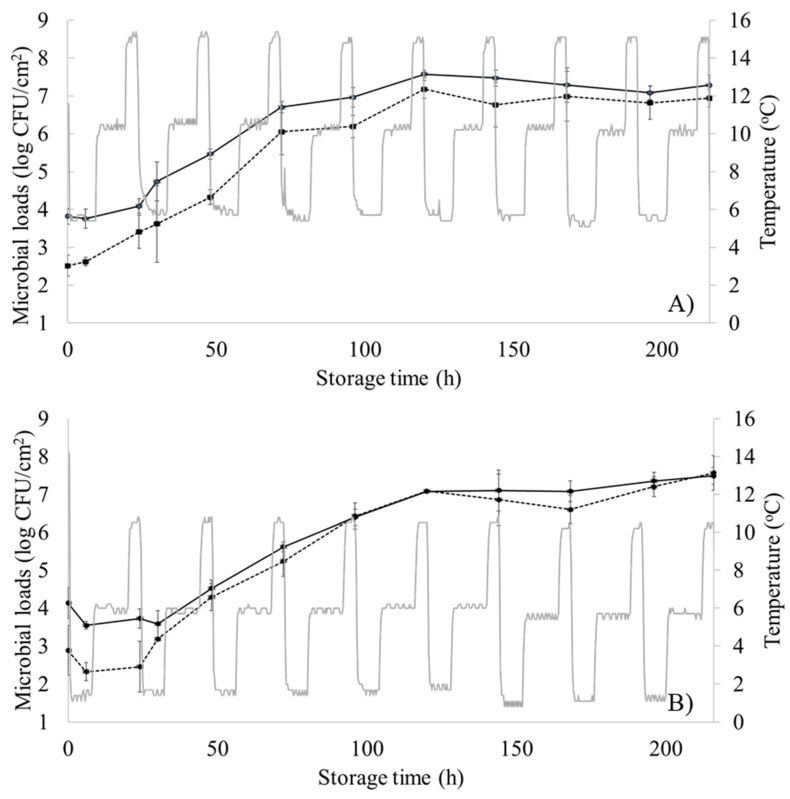
Changes in the population (log CFU/cm^2^) of total viable counts (TVCs) (solid line) and *Pseudomonas* spp. (dashed line) in chicken thigh samples stored under periodically changing temperature conditions. (**A**) Profile 1 = 12 h at 5 °C, 8 h at 10 °C, and 4 h at 15 °C; (**B**) Profile 2 = 12 h at 0 °C, 8 h at 5 °C, and 4 h at 10 °C. Data points are mean values of triplicate samples ± standard deviation.

Moreover, the microbiological results from the two dynamic temperature profiles are shown in Figure 3. The initial TVCs and *Pseudomonas* spp. counts were 3.82 ± 0.21 log CFU/cm^2^ and 2.51 ± 0.28 log CFU/cm^2^, respectively (first dynamic temperature profile, Figure 3A), and 4.13 ± 0.40 log CFU/cm^2^ and 2.87 ± 0.65 log CFU/cm^2^, respectively (second dynamic temperature profile, Figure 3B). Stored samples at these dynamic profiles were considered spoiled in 96 h (TVC = 6.96 ± 0.25 log CFU/cm^2^, *Pseudomonas* spp. = 6.19 ± 0.29 log CFU/cm^2^) for the first dynamic profile and in 120 h (TVC = 7.08 ± 0.01 log CFU/cm^2^, *Pseudomonas* spp. = 7.05 ± 0.03 log CFU/cm^2^) for the second dynamic profile. This one-day delay of spoilage could be attributed to the different metabolic footprint of chicken samples due to temperature alterations affecting thus microbial growth [55,56]. Statistical analysis for the microbiological results (one-way ANOVA via MATLAB 2012a software (The MathWorks, Inc., Natick, MA, USA)) is available in Tables S1 and S2.

More detailed information about chicken thigh fillets spoilage was derived by sensory evaluation, where 56.9% of the samples were scored above 2 and considered spoiled. Samples stored at 0 °C were considered acceptable until 240 h of storage, while samples stored at 30 and 35 °C were evaluated as spoiled after 6 and 12 h, respectively. In addition, deterioration of odor due to spoilage was evident in 96 h at 5 °C, 48 h at 10 °C, and 24 h at 15, 20, and 25 °C. The correlation of sensory scores to samples temperature and TVCs populations is provided at Table 1. TVCs values above 6.99 log CFU/cm^2^ corresponded to samples rated with an average score greater than 2, similarly to other studies where spoilage threshold was established at 7.0 log CFU/cm^2^ for poultry [53]. Based on this criterion, samples were assigned in two quality classes, namely fresh (score < 2) or spoiled (score ≥ 2), and were further employed in the development of classification models.

### 3.2. Correlation of Microbiological Data to Spectral Information

**Figure 4:**
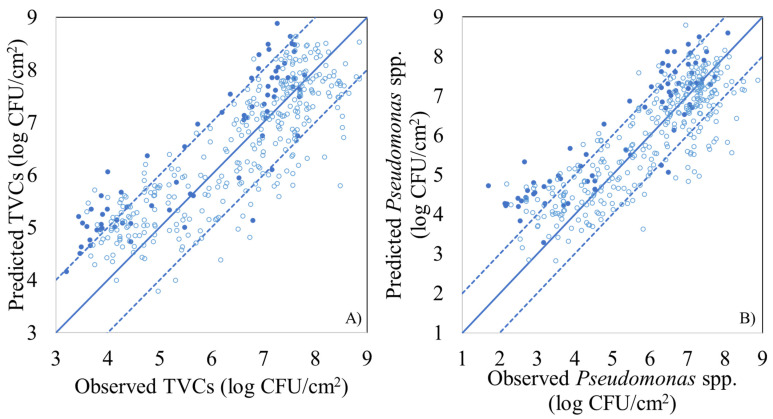
Predicted versus observed TVCs (**A**) and *Pseudomonas* spp. (**B**) counts by the PLS-R models, based on MSI data for FCV (open symbols) and prediction (solid symbols). Solid line represents the line of equity (y = x) and dashed lines indicate ± 1.0 log unit area.

PLS-R model parameters (slope and offset) and performance metrics (r, RMSE), for the estimation of the population of TVCs and *Pseudomonas* spp. using MSI spectral data, are presented in Table 2, for model calibration, full cross validation, and external validation (prediction). For TVCs, the calculated values of RMSE and r during model calibration and cross validation were 0.730 and 0.779 log CFU/cm^2^, as well as 0.861 and 0.840, respectively, whereas the respective values for external validation were 0.987 log CFU/cm^2^ and 0.895, respectively. The performance of the PLS-R model was also graphically illustrated by the comparison of the observed vs. predicted TVCs (Figure 4A). Predicted values were mostly located within the area of ±1.0 log CFU/cm^2^, which is considered microbiologically acceptable, while an overestimation for low counts (below 4.0 log CFU/cm^2^) was evident. Regarding PLS-R model assessing *Pseudomonas* spp. counts via MSI data, RMSE and r values were 0.828 log CFU/cm^2^ and 0.853, respectively, for calibration, while for full cross validation they were 0.886 log CFU/cm^2^ and 0.830, respectively. For external validation (prediction) of *Pseudomonas* spp. counts, RMSE and r values were estimated at 1.215 log CFU/cm^2^ and 0.904 respectively. Nevertheless, the prediction of *Pseudomonas* spp. counts demonstrated deviations (overestimation) from the ± 1.0 log CFU/cm^2^ area, especially for samples with *Pseudomonas* spp. loads lower than 4.0 log CFU/cm^2^ (Figure 4B).

**Figure 5:**
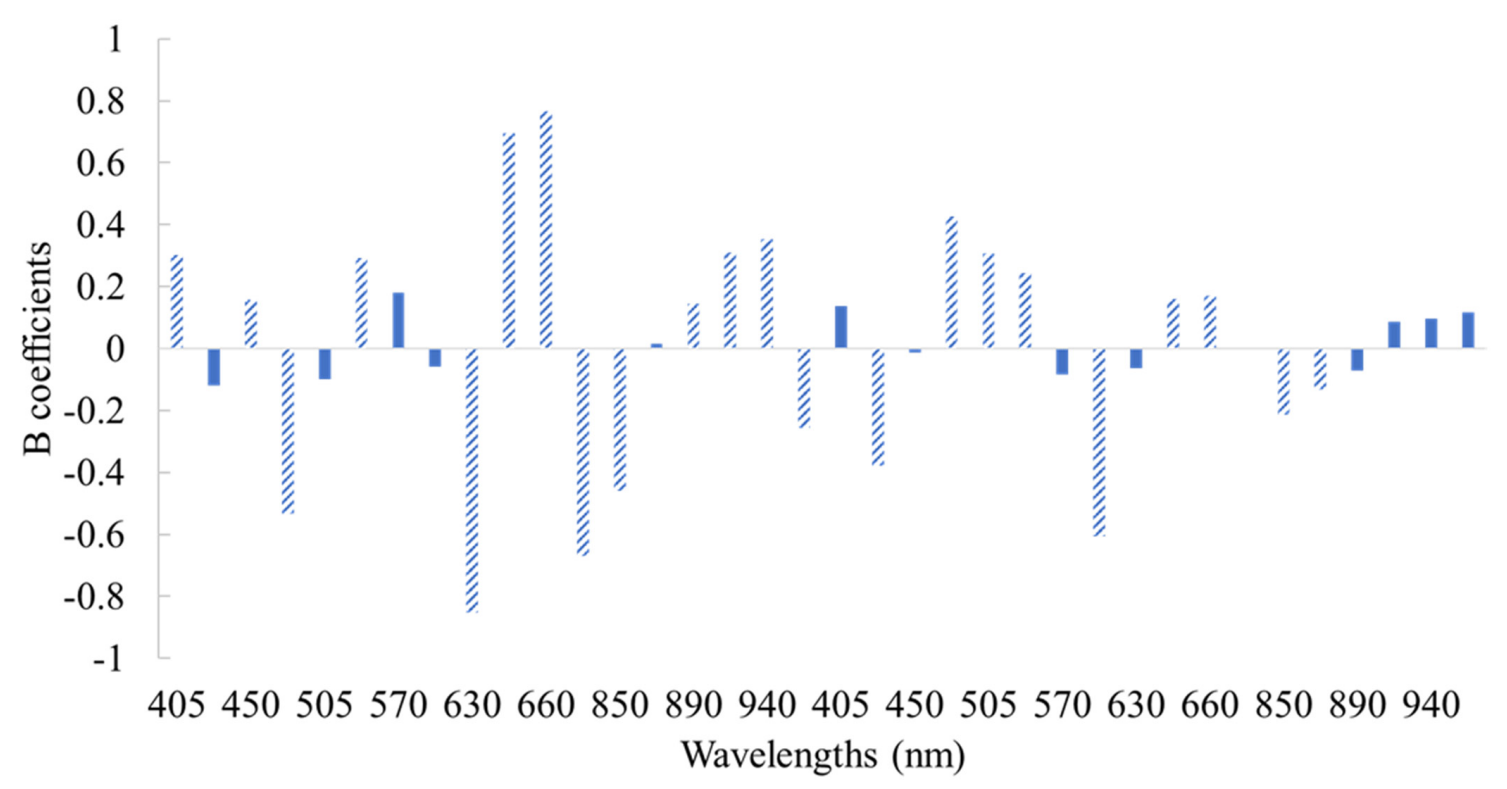
Beta (B) coefficient values of the PLS-R model developed on MSI spectral data for chicken thigh fillets. Shaded bars indicate important variables (mean intensity and standard deviation of pixels from each wavelength).

The important wavelengths contributing to the prediction of the selected microbial groups were obtained according to PLS-R beta coefficients (B), derived by the Unscrambler software and Marten’s Uncertainty test (Figure 5). The wavelengths 630, 645, 660, 700, and 850 nm were identified as significant (b coefficient greater than 0.2) for determining TVCs counts on the surface of chicken thigh. The significant contribution of the wavelength range 630–700 nm for the determination of meat and poultry spoilage has been reported in previous studies, and could be linked to myoglobin, metmyoglobin, deoxymyoglobin or oxymyoglobin [11,20]. According to the B regression coefficients of the PLS-R models, the quantitative equations for the estimation of TVCs and *Pseudomonas* spp. counts via MSI application could be described as follows:

Y_TVCs_= 5.983 + 0.303 × X_mean,405nm_ + 0.158 × X_mean,450nm_ − 0.532 × X_mean,470nm_ + 0.292 × X_mean,525nm_ − 0.853 × X_mean,630nm_ + 0.695 × X_mean,645nm_ + 0.767× X_mean,660nm_ − 0.670 × X_mean,700nm_ − 0.460 × Χ_mean,850nm_ + 0.145 × X_mean,890nm_ + 0.309 × X_mean,910nm_ + 0.352 × X_mean,940nm_ − 0.255 × X_mean,970nm_ − 0.377× X_SD,435nm_ + 0.426 × X_SD,470nm_ + 0.308 × X_SD,505nm_+ 0.244× X_SD,525nm_ − 0.607× X_SD,590nm_ + 0.160× X_SD,645nm_ + 0.171 × X_SD,660nm_ − 0.212 × X_SD,850nm_ − 0.132× X_SD,870nm_ (1)

Y*_Pseudomonas_*_spp. counts_ = 5.416 + 0.204 × X_mean,405nm_ + 0.308 × X_mean,450nm_ − 0.745 × X_mean,470nm_ + 0.326 × X_mean,525nm_ − 1.020 × X_mean,630nm_ + 0.802 × X_mean,645nm_ + 0.885× X_mean,660nm_ − 0.766 × X_mean,700nm_ − 0.500 × Χ_mean,850nm_ + 0.332 × X_mean,910nm_ + 0.422 × X_mean,940nm_ − 0.344 × X_mean,970nm_ − 0.602× X_SD,435nm_ + 0.501 × X_SD,470nm_ + 0.367 × X_SD,505nm_+ 0.272× X_SD,525nm_ − 0.679× X_SD,590nm_ + 0.244× X_SD,645nm_ + 0.222 × X_SD,660nm_ − 0.321 × X_SD,850nm_ − 0.204× X_SD,870nm_ - 0.133× X_SD,890nm_ + 0.159 × X_SD,910nm_ + 0.177 × X_SD,970nm_ (2)

In the above equations, the response variable (Y) can be approximated by a linear combination of the values of the predictors (X) through coefficients called regression or B -coefficients. Specifically, Y is the estimated value for TVCs and *Pseudomonas* spp., respectively, whereas X_mean_ and X_SD_ are the mean intensity and the standard deviation of the pixels at the respective wavelength during MSI acquisition, respectively.

**Figure 6:**
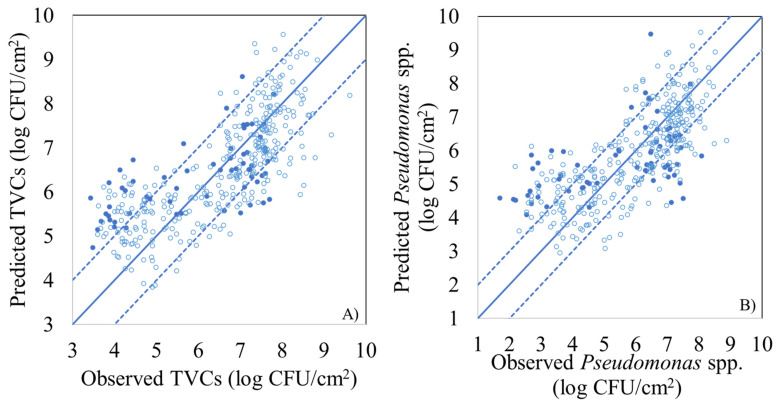
Predicted versus observed TVCs (**A**) and *Pseudomonas* spp. counts (**B**) by the PLS-R models, based on FT-IR data for FCV (open symbols) and prediction (solid symbols). Solid line represents the line of equity (y = x) and dashed lines indicate ± 1.0 log unit area.

**Figure 7:**
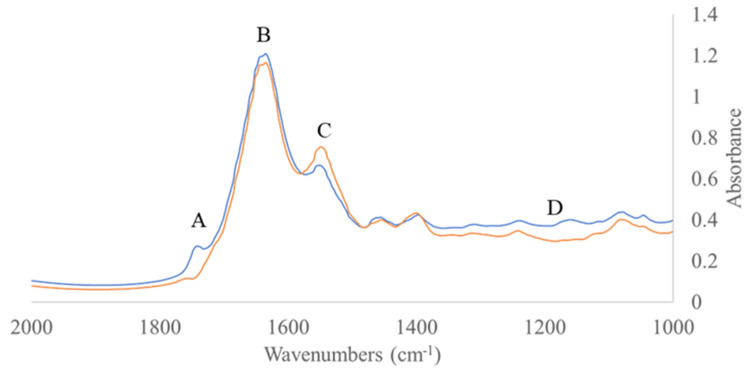
Typical FT-IR spectra in the range of 1000–2000 cm^−1^ (A: 1720–1790 cm^−1^; B: 1630–1690 cm^−1^; C: 1500–1550 cm^−1^ and D: 1300–1100 cm^−1^) collected from chicken thigh fillet stored at 0 °C for 0 h (fresh = blue line) and after 366 h (spoiled = orange line).

**Figure 8:**
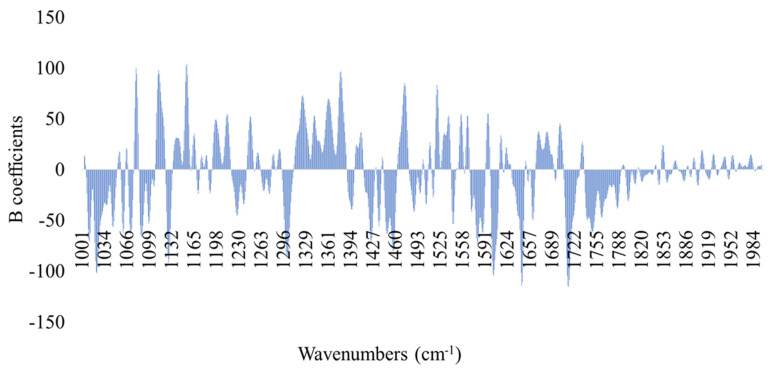
Beta (B) coefficients for PLS-R model developed on FT-IR spectral data for chicken thigh fillets.

Likewise, model performance for the estimation of TVCs and *Pseudomonas* spp. counts via FT-IR spectral data analysis is presented in Table 3. For the TVCs prediction model, RMSE and r values for calibration and full cross validation were 0.734 log CFU/cm^2^ and 0.856, as well as 0.899 log CFU/cm^2^ and 0.781, respectively, while for external validation they were 1.251 log CFU/cm^2^ and 0.583, respectively. Similarly, for the prediction of *Pseudomonas* spp. counts via FT-IR analysis, RMSE and r values were 0.838 log CFU/cm^2^ and 0.849 for calibration, 1.037 log CFU/cm^2^ and 0.762 for full cross validation, and 1.589 log CFU/cm^2^ and 0.514 for external validation, respectively. The performance of the PLS-R models was also graphically verified by the comparison of the observed versus predicted counts of TVCs and *Pseudomonas* spp. (Figure 6), demonstrating an overestimation in the fail-safe zone for samples with TVCs values lower than 4.0 log CFU/cm^2^ (Figure 6A). In contrast, according to Figure 6B, *Pseudomonas* spp. predicted counts deviated from the acceptable limit of ± 1.0 log CFU/cm^2^, presenting both overestimated (for counts < 4.0 log CFU/cm^2^) and underestimated (for counts > 7.0 log CFU/cm^2^) values. In addition, the influence of each wavenumber in the development of the PLS-R models via FT-IR spectroscopy is highlighted by the beta coefficients (Figure 7), as well as by the representative spectra acquisition for fresh (0 h at 0 °C) and spoiled (366 h at 0 °C) samples (Figure 8). Four main regions demonstrated high impact on model development, namely: region A (1720–1790 cm^−1^); region B (1630–1690 cm^−1^); region C (1500–1550 cm^−1^) and region D (1300–1100 cm^−1^). It is well established that these absorption regions are related to the proteolytic activity of microbiota and the formation of biofilms, and more specifically of *Pseudomonas* spp. during spoilage of chicken breast [21,22,24,57].

### 3.3. Classification Models for the Assessment of Spoilage

The performance of the selected models to classify the samples in the respective quality classes (fresh or spoiled) through MSI spectral data is demonstrated by the confusion matrix (Table 4) for LDA, QDA, SVM, and QSVM. For the LDA model, 219 out of 330 samples and 49 out of 72 samples were correctly classified in both quality classes during model development (FCV) and prediction, respectively, providing overall accuracy of 66.4% and 68.1%. During FCV process, sensitivity and specificity were 59.4% and 73.3%, respectively, whereas for model prediction the calculated sensitivity and specificity were 76.0% and 63.8%, respectively. For QDA model, 214 out of 330 samples (overall accuracy 64.8%) and 50 out of 72 samples (overall accuracy 69.4%) were classified in the correct class for model FCV and prediction, respectively. Moreover, sensitivity and specificity were estimated at 57.6% and 72.8%, respectively, for model FCV and at 73.3% and 66.7%, respectively, for the model prediction. It is notable that improved results were obtained by the application of SVM model where 301 out of 330 samples (overall accuracy 91.2%) and 68 out of 72 samples (overall accuracy 94.4%) were correctly classified in the respective quality class during model development (FCV) and prediction, respectively. In addition, for SVM model sensitivity and specificity, percentages exhibited their highest values at 94.4% during external validation. Likewise, for QSVM implementation, 287 from 330 samples and 66 from 72 samples were efficiently identified during model development and prediction, with an overall accuracy of 87.0% and 91.7%, respectively. For this model, sensitivity and specificity percentages were calculated at 83.7% and 89.6% for model FCV, while for external validation the estimated values were 94.1% and 89.5%, respectively.

Regarding FT-IR classification models (Table 5), the LDA model classified correctly 240 out of 328 samples and 44 out of 63 samples during model FCV and external validation, respectively, with overall accuracy reaching 73.2% and 69.8%, respectively. Sensitivity and specificity percentages were 64.1% and 84.7% for model development, whereas for external validation these performance metrics were 70.4% and 69.4%, respectively. For QDA method, 216 out of 328 samples and 45 out of 63 samples were classified at their proper quality group during FCV and external validation, respectively. QDA model enhanced performance against the remaining three models was underlined by its ability to classify fresh samples from an independent validation data set with sensitivity and specificity values of 70.0% and 72.7%, respectively. For QSVM model, 284 out of 328 samples were correctly classified during model development (overall accuracy 86.6%, sensitivity 82.4%, specificity 90.0%), whereas only 38 from 63 samples were located in their correct class during model prediction (overall accuracy 60.3%, sensitivity 55.8%, specificity 70%). Finally, for SVM model 287 out of 328 samples were accurately classified during model FCV (overall accuracy 87.5%, sensitivity 90.7%, specificity 85.1%), whereas during model prediction 44 out of 63 samples were classified correctly in their respective quality class (overall accuracy 69.8%, sensitivity 63.4%, specificity 81.8%).

It needs to be noted that MSI-SVM and FT-IR-QDA combinations could not only efficiently classify samples in their correct quality class, with overall accuracy of 94.4% and 71.4%, respectively, but, simultaneously, the misclassified samples were equally distributed in the safe and in the dangerous side, with specificity reaching 94.4% and 72.7%, respectively. Another interesting finding from MSI-SVM model was the low difference in the overall accuracy percentages (91.2% vs. 94.4%) observed between model FCV and prediction, indicating robust model performance. Furthermore, the same trend was observed for sensitivity and specificity (94.0% in both cases). Previous researchers reported that SVMs could result in the development of robust regression and classification models for poultry products [31,58]. SVM and QSVM models were more suitable for MSI spectral data, with SVM linear classifiers presenting the best separation of data’s hyperplane [31]. In contrast, probability parametric LDA and QDA models which assume that each class could be described as a multivariate normal distribution [29,31], exhibited better discrimination of classes for FT-IR data. This is in good agreement with other studies, where LDA was proposed as a supervised multivariate classification method in FT-IR spectroscopic analysis of meat samples [25]. Even though data matrices from MSI and especially FT-IR presented high dimensionality, there was no evident class imbalance according to the prediction performance of all developed models (Table 4 and Table 5).

## 4. Conclusions

The findings of this research indicated that MSI spectral data combined with PLS-R could satisfactorily predict TVC and *Pseudomonas* spp. counts on the surface of chicken thigh fillets regardless of storage temperature and batch variation. Similarly, satisfactory performance was obtained by the implementation of the classification models, where MSI spectral data coupled to SVM model achieved the most accurate identification of quality classes (fresh or spoiled) among samples. Moreover, QDA was considered the most suitable classification model applied to FT-IR measurements. The generalization of the developed models was ascertained by the utilization of chicken samples stored at two dynamic temperature profiles simulating conditions of the distribution chain. Nevertheless, additional measurements and continuous information feedback (different seasons, packaging conditions, etc.) could improve the performance of the aforementioned models and thus result in more successful assessment of the quality of poultry products.

## Figures and Tables

**Table 1:** Sensory scores and TVCs counts for chicken thigh samples corresponding to the sensory rejection time at each storage temperature.

Temperature (°C)	Storage Time (h)	Odor	TVCs (log CFU/cm^2^)
0	240	2.5	6.99
5	96	2.3	7.08
10	48	2.3	6.90
15	24	2.1	7.46
20	24	2.5	7.40
25	24	2.9	8.22
30	6	2.1	5.1
35	12	2.2	6.84
TVCs average (log CFU/cm^2^)	6.99		

**Table 2:** Performance metrics of the developed PLS-R models estimating TVCs and *Pseudomonas* spp. counts of chicken thigh samples via MSI spectral data analysis.

TVCs	*n*	LVs	Slope	Offset	r	RMSE
Calibration Full Cross Validation Prediction	330	10	0.741	1.684	0.861	0.730
	330	10	0.726	1.787	0.840	0.779
	72		0.774	2.023	0.895	0.987
*Pseudomonas* spp.	*n*	LVs	slope	offset	r	RMSE
Calibration Full Cross Validation Prediction	330	10	0.727	1.615	0.853	0.828
	330	10	0.711	1.714	0.830	0.886
	72		0.702	2.441	0.904	1.215

*n*: Number of samples, LVs: Latent variables, r: Correlation coefficient, RMSE: Root mean squared error.

**Table 3:** Performance metrics of the developed PLS-R models estimating TVCs and *Pseudomonas* spp. counts of chicken thigh samples via FT-IR spectral data analysis.

TVCs	*n*	LVs	Slope	Offset	r	RMSE
Calibration Full Cross Validation Prediction	328	10	0.732	1.747	0.856	0.734
	328	10	0.678	2.115	0.781	0.899
	63		0.367	4.192	0.583	1.251
*Pseudomonas* spp.	*n*	LVs	slope	offset	r	RMSE
Calibration Full Cross Validation Prediction	328	10	0.719	1.669	0.849	0.838
	328	10	0.660	2.033	0.762	1.037
	63		0.282	4.152	0.514	1.589

*n*: Number of samples, LVs: Latent variables, r: Correlation coefficient, RMSE: Root mean squared error.

**Table 4:** Confusion matrix and performance indexes of the developed classification models (LDA, QDA, SVM, QSVM) regarding sensory quality discrimination of chicken thigh samples based on MSI spectral data.

Model	Procedure	O/P	Fresh	Spoiled	Overall	Sensitivity (%)	Specificity (%)
LDA	FCV	Fresh	98	67	330	59.4	73.3
		Spoiled	44	121		73.3	59.4
		Overall accuracy (%)		66.4			
	Prediction	Fresh	19	6	72	76.0	63.8
		Spoiled	17	30		63.8	76.0
		Overall accuracy (%)		68.1			
QDA	**Procedure**	**O/P**	**Fresh**	**Spoiled**	**Overall**	**Sensitivity (%)**	**Specificity (%)**
	FCV	Fresh	99	73	330	57.6	72.8
		Spoiled	43	115		72.8	57.6
		Overall accuracy (%)		64.8			
	Prediction	Fresh	22	8	72	73.3	66.7
		Spoiled	14	28		66.7	73.3
		Overall accuracy (%)		69.4			
SVM	**Procedure**	**O/P**	**Fresh**	**Spoiled**	**Overall**	**Sensitivity (%)**	**Specificity (%)**
	FCV	Fresh	130	17	330	88.4	93.4
		Spoiled	12	171		93.4	88.4
		Overall accuracy (%)		91.2			
	Prediction	Fresh	34	2	72	94.4	94.4
		Spoiled	2	34		94.4	94.4
		Overall accuracy (%)		94.4			
QSVM	**Procedure**	**O/P**	**Fresh**	**Spoiled**	**Overall**	**Sensitivity (%)**	**Specificity (%)**
	FCV	Fresh	123	24	330	83.7	89.6
		Spoiled	19	164		89.6	83.7
		Overall accuracy (%)		87.0			
	Prediction	Fresh	32	2	72	94.1	89.5
		Spoiled	4	34		89.5	94.1
		Overall accuracy (%)		91.7			

**Table 5:** Confusion matrix and performance indexes of the developed classification models (LDA, QDA, SVM, QSVM) regarding sensory quality discrimination of chicken thigh samples based on FT-IR spectral data.

Model	Procedure	O/P	Fresh	Spoiled	Overall	Sensitivity (%)	Specificity (%)
LDA	FCV	Fresh	118	66	328	64.1	84.7
		Spoiled	22	122		84.7	64.1
		Overall accuracy (%)		73.2			
	Prediction	Fresh	19	8	63	70.4	69.4
		Spoiled	11	25		69.4	70.4
		Overall accuracy (%)		69.8			
QDA	**Procedure**	**O/P**	**Fresh**	**Spoiled**	**Overall**	**Sensitivity (%)**	**Specificity (%)**
	FCV	Fresh	118	87	328	57.6	79.7
		Spoiled	25	98		79.7	57.6
		Overall accuracy (%)		65.9			
	Prediction	Fresh	21	9	63	70.0	72.7
		Spoiled	9	24		72.7	70
		Overall accuracy (%)		71.4			
SVM	**Procedure**	**O/P**	**Fresh**	**Spoiled**	**Overall**	**Sensitivity (%)**	**Specificity (%)**
	FCV	Fresh	127	13	328	90.7	85.1
		Spoiled	28	160		85.1	90.7
		Overall accuracy (%)		87.5			
	Prediction	Fresh	26	15	63	63.4	81.8
		Spoiled	4	18		81.8	63.4
		Overall accuracy (%)		69.8			
QSVM	**Procedure**	**O/P**	**Fresh**	**Spoiled**	**Overall**	**Sensitivity (%)**	**Specificity (%)**
	FCV	Fresh	122	26	328	82.4	90.0
		Spoiled	18	162		90	82.4
		Overall accuracy (%)		86.6			
	Prediction	Fresh	24	19	63	55.8	70.0
		Spoiled	6	14		70.0	55.8
		Overall accuracy (%)		60.3			

## Data Availability

The datasets generated for this study are available on request to the corresponding author.

## Supplementary Materials

The following are available online at https://www.mdpi.com/article/10.3390/foods10112723/s1, Table S1: One way ANOVA for the TVC of chicken thigh fillet samples at each isothermal storage temperature and dynamic temperature scenarios, Table S2: One way ANOVA for Pseudomonas spp. counts of chicken thigh fillet samples at each isothermal storage temperature and dynamic temperature scenarios.
