# Supplementary material for: Microbiological Quality Assessment of Chicken Thigh Fillets Using Spectroscopic Sensors and Multivariate Data Analysis
Source: Foods. 2021 Nov 7;10(11):2723. doi: 10.3390/foods10112723 (PMC8624579; doi:10.3390/foods10112723)
Supplement: Supplementary file 1 [file foods-10-02723-s001.zip › foods-1431846-supplementary.pdf]

Table S1: One way ANOVA for the TVCs of chicken thigh fillet samples at each isothermal storage temperature and dynamic temperature scenarios.

Temperature (°C)	Source	SS	df	MS	F	p-value
**15**	**Columns**	0.652	3	0.21743	0.09	0.9672
	**Error**	100.915	40	2.52289		
	**Total**	11.568	43			
**10**	**Columns**	0.5359	3	0.17862	0.08	0.9691
	**Error**	95.0444	44	2.1601		
	**Total**	95.5803	47			
**5**	**Columns**	0.475	3	0.15833	0.08	0.9713
	**Error**	96.6625	48	2.0138		
	**Total**	97.1375	51			
**0**	**Columns**	0.4734	3	0.15782	0.1	0.9588
	**Error**	99.3133	64	1.55177		
	**Total**	99.7867	67			
**20**	**Columns**	0.208	3	0.6923	0.02	0.9957
	**Error**	129.93	40	3.24826		
	**Total**	130.138	43			
**25**	**Columns**	0.043	3	0.01431	0	0.9996
	**Error**	118.588	36	3.2941		
	**Total**	118.631	39			
**30**	**Columns**	0.0816	3	0.0272	0.01	0.9986
	**Error**	98.911	36	2.74753		
	**Total**	98.9926	39			
**35**	**Columns**	0.833	3	0.27767	0.1	0.957
	**Error**	85.1931	32	2.66228		
	**Total**	86.0261	35			
**Dynamic 1**	**Columns**	0.0029	2	0.00143	0	0.9993
	**Error**	63.5467	30	2.11822		
	**Total**	63.5495	32			
**Dynamic 2**	**Columns**	0.0703	2	0.03515	0.01	0.9871
	**Error**	81.139	30	2.70463		
	**Total**	81.2093	32			
**Dynamic 1 and 2**	**Columns**	3.385	5	0.67696	0.28	0.9219
	**Error**	144.686	60	2.41143		
	**Total**	148.07	65			

Table S2: One way ANOVA for *Pseudomonas* spp. counts of chicken thigh fillet samples at each isothermal storage temperature and dynamic temperature scenarios.

Temperature (°C)	Source	SS	df	MS	F	p-value
**15**	Columns	0.216	3	0.07205	0.02	0.9948
	Error	118.162	40	2.95404		
	Total	118.378	43			
**10**	Columns	0.107	3	0.03552	0.01	0.9982
	Error	130.044	44	2.9554		
	Total	130.15	47			
**5**	Columns	0.736	3	0.24524	0.1	0.9569
	Error	103.108	44	2.34336		
	Total	103.844	47			
**0**	Columns	2.069	3	0.68962	0.4	0.7545
	Error	110.749	64	1.73045		
	Total	112.818	67			
**20**	Columns	0.756	3	0.25205	0.06	0.9808
	Error	169.741	40	4.24353		
	Total	170.497	43			
**25**	Columns	1.068	3	0.35598	0.09	0.9643
	Error	140.257	36	3.89602		
	Total	141.325	39			
**30**	Columns	3.4919	3	1.16398	0.44	0.7251
	Error	95.0259	36	2.63961		
	Total	98.5179	39			
**35**	Columns	0.8236	3	0.27453	0.14	0.9353
	Error	62.7508	32	1.96096		
	Total	63.5744	35			
**Dynamic 1**	Columns	0.0427	2	0.02133	0.01	0.9908
	Error	68.9284	30	2.29761		
	Total	68.971	32			
**Dynamic 2**	Columns	0.0775	2	0.03877	0.01	0.9873
	Error	91.2975	30	3.04325		
	Total	91.3751	32			
**Dynamic 1 and 2**	Columns	0.901	5	0.1802	0.07	0.9967
	Error	160.226	60	2.67043		
	Total	161.127	61			
